# Opium and bladder cancer: A systematic review and meta-analysis of the odds ratios for opium use and the risk of bladder cancer

**DOI:** 10.1371/journal.pone.0178527

**Published:** 2017-06-06

**Authors:** Mahdi Afshari, Ghasem Janbabaei, Mohammad Amin Bahrami, Mahmood Moosazadeh

**Affiliations:** 1 Department of Community Medicine, Zabol University of Medical Sciences, Zabol, Iran; 2 Gastrointestinal Cancer Research Center, Faculty of Medicine, Mazandaran University of Medical Sciences, Sari, Iran; 3 Healthcare Management Department, Shahid Sadoughi University of Medical Sciences, Yazd, Iran; 4 Health Sciences Research Center, Addiction Institute, Mazandaran University of Medical Sciences, Sari, Iran; National Cancer Center, JAPAN

## Abstract

**Objective:**

The association between opium use and bladder cancer has been investigated in many studies, with varying reporting results reported. This study aims to estimate the total odds ratio for the association between bladder cancer and opium consumption using meta-analysis.

**Methods:**

The study was designed according to PRISMA guidelines. Two independent researchers searched for the relevant studies using PubMed, Web of Science, Scopus, OVID, Embase, and Google Scholar. After systematic screening of the studies identified during the first step, Cochrane risk of bias tool was determined for the selected studies. The case-control and the cohort studies were investigated to assess risk of bladder cancer due to opium use. In addition, the cross-sectional studies were analysed separately to assess frequency of opium consumption. These estimates were combined using the inverse variance method. Fixed or random effect models were applied to combine the point odds ratios. The heterogeneity between the primary results was assessed using the Cochran test and I-square index. The suspected factors for heterogeneity were investigated using meta-regression models. An Egger test was conducted to identify any probable publication bias. Forest plots illustrated the point and pooled estimates. All analyses were performed using Stata version 14 software and RevMan version 5.3.

**Results:**

We included 17 primary studies (11 case-control, one cohort and five cross-sectional) in the final meta-analysis. The total odds ratios (95% confidence intervals) for developing bladder cancer by opium use alone, and concurrent use of opium and cigarettes were estimated as 3.85 (3.05–4.87) and 5.7 (1.9–16.3) respectively. The odds ratio (95% confidence interval) for opium use with or without cigarette smoking was estimated as 5.3 (3.6–7.7).

**Conclusion:**

This meta-analysis showed that opium use similar to cigarette smoking and maybe with similar mechanisms can be a risk factor for bladder cancer. It is therefore expected to be a risk factor for other cancers.

## Introduction

Bladder cancer is responsible for more than 7% of neoplasms, especially among men [1]. Annually, more than 12 million cases of bladder cancer are reported worldwide [2]), and it is among the eleven top ranked incident cancers in the world [3]. The incidence and mortality rates of bladder cancer have increased in Eastern European and developing countries in recent years [3].

Several risk factors are reported to be responsible for developing bladder cancer such as high body mass index [4], genetic factors [5], occupational hazards [6–7], environment and nutrition [8], and behavioral factors [7, 9–11].

Behavioral factors, especially cigarette smoking and opium addiction, have been mentioned in previous studies as the most important risk factors for many cancers including bladder cancer [12, 13]. Although many studies focused on the relationship between this cancer and opium consumption, the independent effect of this factor has been ignored. Based on the results of the above-mentioned studies, behavior/status of opium users can strongly distort or modify the association between opium use and bladder cancer, and should be taken into consideration when assessing the carcinogenic effect of opium addiction. In addition, almost all the previous studies investigating this relationship are primary studies with limited sample sizes and powers as well as contradictory results. Moreover, in the review study conducted by Kamangar et al. [12], the results were not combined using a meta-analysis method. Moreover, the total effect of opium on developing bladder cancer controlling for the confounding influence of cigarette smoking has not been estimated elsewhere.

This study aimed to combine the results of these primary studies using a meta-analysis to estimate a more precise association between opium addiction and bladder cancer.

## Methods

The results were presented using the PRISMA statement method [14]. The study was conducted according to a pre-designed protocol [15].

### Inclusion criteria

1) Studies with bladder cancer as the outcome of interest (cases were patients with bladder cancer). 2) Cases were compared with a control group of either opium use only, or opium use plus cigarette smoking. 3) Studies carried out up to October 30, 2016. 4) Case-control, cohort, and cross- sectional studies. 5) Studies written in English or Persian. 6) Studies investigating the frequency of cigarette smoking among patients with bladder cancer. Meanwhile PICO system is designed as follows; P: opium users I: not applicated for this study, C: non-opium users O: bladder cancer.

### Databanks and search strategy

Several databanks such as PubMed, Science direct, Scopus, Embase, Ovid, Google Scholar, and Persian databases such as Magiran(http://www.magiran.com/) and SID(http://sid.ir/) were searched using MESH headings and relevant keywords (neoplasms, cancer, bladder, opium, patient, case-control, cohort, and cross-sectional). Two independent researchers performed all searches. A third researcher was also involved as decision maker for any disagreements. All references were investigated to increase the search sensitivity. The search was managed using Endnote software.

### Study selection

Two independent researchers selected relevant studies based on the inclusion criteria. Screening was performed after restriction of the search strategy and exclusion of duplicates. Irrelevant papers were identified during the investigation of titles, abstracts, and full texts respectively. The agreement between the selection results of the researchers was assessed based on kappa statistics suggested in the Landis & Koch guidelines [16]. The agreement was considered as slight (kappa between 0–0.20), fair (kappa between 0.21–0.40), moderate (kappa between 0.41–0.60), substantial (kappa between 0.61–0.80), and perfect (kappa more than 0.80).

### Quality assessment

The Cochrane risk of bias tool [17–18] was used for quality assessment of the primary studies. Two independent researchers performed the quality assessment and any disagreement was resolved by a third researcher. The risk of bias was assessed from the viewpoints of selection bias, performance bias, detection bias, attrition bias, and other sources of bias (e.g. bias of study design and extreme baseline imbalance).

The overall risk of bias was categorized as zero (high risk), two (low risk), and one (unclear risk). In addition, the STROBE checklist [19] was applied for quality assessment of the cross-sectional studies. This checklist contains 22 items investigating the different aspects of methodology such as objects, sampling, designs, tools, statistical tests, variables, and presentations. The cross-sectional studies were assigned ratings from zero to 44 according to the STROBE assessment, including low quality (less than 15.5), moderate quality (15.5–29.5), and high quality (30–44). Low quality studies were excluded from the meta-analysis.

### Data extraction

The required information was extracted and entered into Excel spreadsheets. The data extraction was carried out by two researchers, while a third researcher resolved any disputes between the two main researchers. The data collected were first author’s name, date and place of the study conduction, sample size and sampling methods for cases/controls or exposed/unexposed groups, age and sex distribution of participants, number of opium users and cigarette smokers in each group, adjusted odds ratios and 95% confidence intervals. In addition, required information such as first author name, date and place of the study conduction, number of patients suffering from bladder cancer and prevalence of opium use among these patients were extracted from each cross-sectional study.

### Data analysis

The analyses were performed using Stata version 14 (Stata Corp, College Station, TX, USA) and the Cochrane Collaboration Review Manager software (RevMan version 5.3). In case control and cohort studies, contingency tables were designed for each study and the primary results were combined using the inverse variance method. In cross-sectional studies, the standard error of the frequency of opium use was calculated based on the binomial distribution formula. Random or fixed effect models were applied to combine the results. The heterogeneity between the primary results was assessed using the Cochran’s Q test and I-squared indicator [20]. Forest plots were used to present the pooled estimates of odds ratios and the 95% confidence intervals. To assess the degree of publication bias, an Egger test was applied considering a p-value of less than 0.1 as statistically significant. The suspected factors for heterogeneity were investigated using meta-regression models.

## Results

### Study selection

**Fig 1:**
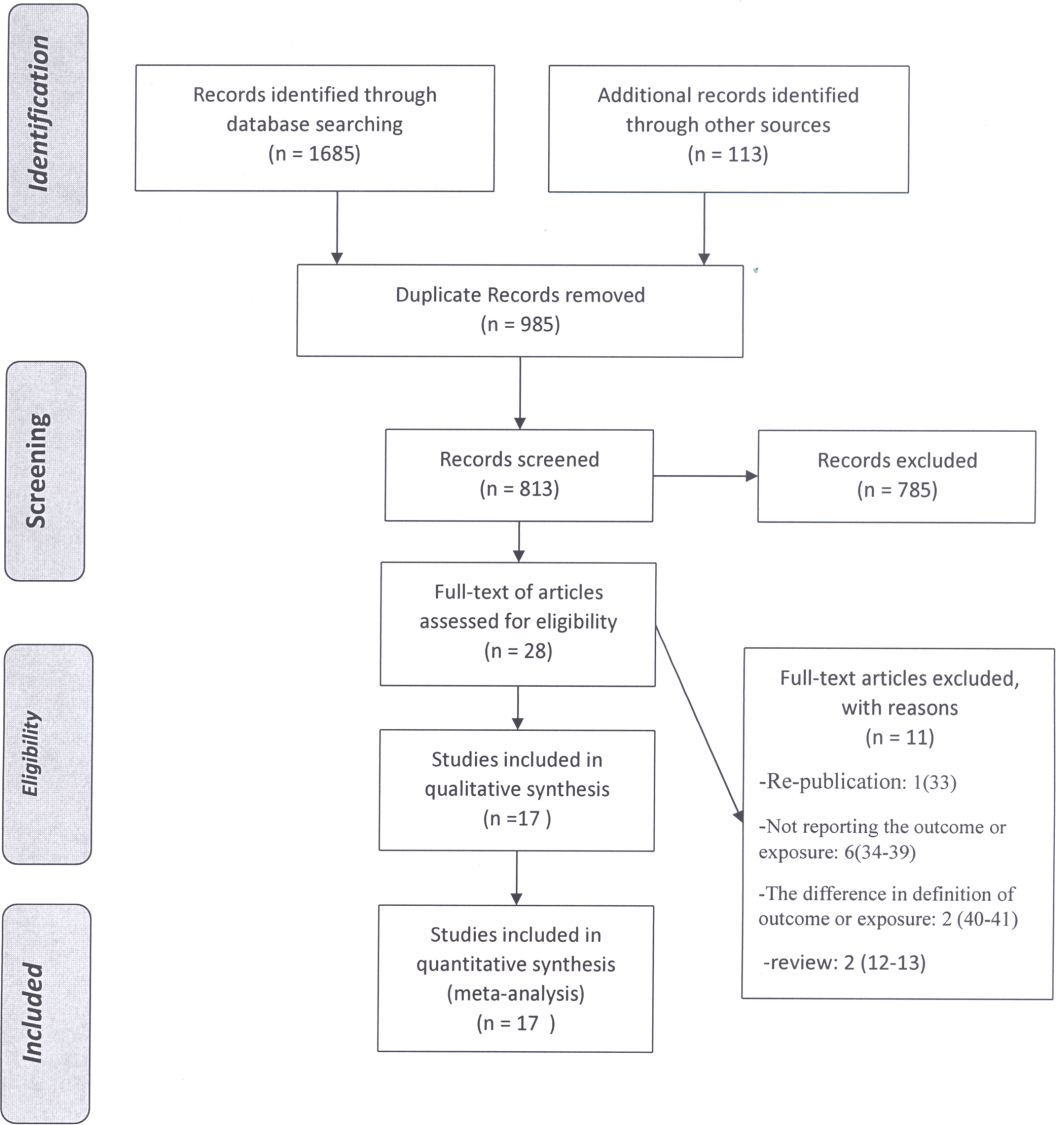
Flowchart of study selection.

We identified 1798 papers during a comprehensive search in the mentioned databanks. Of them, 985 papers were duplicated in different databanks and were excluded. The remained 813 papers were screened based on their titles and abstracts and 785 irrelevant articles were removed. During a full text review of 28 papers [7, 9–13, 21–42], 11 papers were excluded [12–13, 34–42]. Finally, 17 studies [7, 9–11, 21–33] were entered into the quality assessment and meta-analysis (Fig 1). The kappa statistic was 0.77 indicating substantial agreement between the researchers.

### Characteristics of the primary studies

We entered 11 case control, one cohort, and five cross- sectional studies into the meta-analysis. Of those, 12 studies investigated the association between opium and bladder cancer, while five reported the frequency of opium consumption among patients with bladder cancer. All studies were carried out in Iran. Analytic studies (case controls and cohorts) were performed before 2000 (three studies) and after 2004 (nine studies). The cross-sectional studies were performed between 2002 and 2013. The source of control groups was reported in 10 studies including hospitals (seven studies) and neighbors (three studies). Controls were defined in 11 studies including healthy individuals (four studies) and patients (seven studies). Cases and controls were matched in all studies (Tables 1–3).

**Table 1:** Characteristics of the primary analytic studies regarding the association between bladder cancer and opium consumption.

Id	First author	Publication year	Country	Source of control	Definition of control	Variables matched
1	Akbari	2015	Iran(Shiraz)	Community (neighborhood)	Healthy individuals	Age, Gender, Residence area
2	Aliramaji	2015	Iran(Mazandaran)	Hospital	Patients for the investigation of gall bladder stone. In addition, the control group had no genitourinary symptoms	Age, Gender
3	Asgari	2004	Iran (Tehran)	Hospital	patients with benign prostatic hyperplasia	Age, both group included male
4	Ghadimi	2015	Iran (Kurdistan)	Hospital	Patient who referred to a specialized clinic	Age, Gender, Residence
5	Hosseini	2010	Iran (Tehran)		genetically unrelated healthy subjects without a history of cancer	age, Gender
6	Ketabchi	2005	Iran (Kerman)	Hospital	tumor-free patients	age, Gender
7	Lotfi	2016	Iran (Yazd)	Community (neighborhood)	Healthy individuals	age, sex and location
8	Nourbakhsh	2006	Iran (Tehran)	Hospital	Patient admitted into trauma wards and had no history or presenting signs or symptoms of urinary problems	age, Gender, cigarette smoking
9	Behmard	1981	Iran(Shiraz)	-	-	age, Gender, cigarette smoking
10	Sadeghi	1978	Iran(Shiraz)	Hospital	The controls were selected from the inpatients who had a diagnosis other than cancer, pulmonary disease, or a bladder condition.	Age, Gender
11	Shakhssalim	2010	Iran (Tehran, Khorasan, Khoozestan, Isfahan and East Azarbayjan)	Community (neighborhood)	Healthy individuals	age, Gender
12	Tootonchi	2000	Iran (Isfahan)	Hospital	Patient without hematuria	age, Gender

**Table 2:** Exposure to opium among cases and controls in the primary studies entered into the meta-analysis.

Id	First author	number		number of exposed with only opium consumption		OR adjusted for opium consumption				number of exposed with Opium with or without cigarette		number of simultaneous exposed with opium and cigarette		Dose-response (P-value of association with BC)	
		Cases	Controls	Cases	Controls	adjusted by variables of	OR	Lower	Upper	Cases	Controls	Cases	Controls	amount of abused opium	duration of opium abuse
1	Akbari	198	396	-	-	Nutritional factors, Tobacco use, alcohol	3.9	1.3	12	43	18	-	-	NS^*****^	<0.001
2	Aliramaji	175	175	14	7	-	-	-	-	58	27	44	20	-	<0.001
3	Asgari	52	108	1	0	-	-	-	-	13	5	12	5	-	<0.001
4	Ghadimi	152	152	-	-	Cigarette smoking, Hypertension, BMI, Education, Nephrolithiasis	4.96	1.1	22.9	16	2	-	-	-	-
5	Hosseini	179	179	-	-	age, sex, smoking, history, and family history of cancer	4.6	3.5	6.3	60	7	-	-	-	<0.001
6	Ketabchi	112	130	-	-	-	-	-	-	80	31	-	-	NS	NS
7	Lotfi	199	200	-	-	-	-	-	-	52	21	-	-	-	-
8	Nourbakhsh	255	255	3	0	-	-	-	-	41	12	-	-	-	-
9	Behmard	3500^*^	1750^**^	15^***^	0^****^	-	-	-	-	-	-	-	-	-	-
10	Sadeghi	88	88	1	1	-	-	-	-	44	7	43	6	-	-
11	Shakhssalim	418	494	-	-	Smoking	2.57	1.55	4.26	-	-	-	-	-	-
12	Tootonchi	142	142	-	-	-	-	-	-	16	7	-	-	-	-

*: Exposed with opium

**: Unexposed with opium

***: The number of cases in exposed group

****: the number of cases in unexposed group

*****: Not significant

**Table 3:** Characteristics of the cross sectional studies investigating the frequency of opium consumption among patients with bladder cancer.

Id	First author	Publication year	Country	Age		Male to Female Ratio	Total number of bladder cancer	Frequency (%)		
				Mean	SD			opium consumption with or without smoking	Simultaneously consumption of Opium and cigarette smoking	Only opium consumption
1	Ahmadi	2012	Iran (Mazandaran)	68.01	14.6	7.1	112	19.6	-	-
2	Karbakhsh	2013	Iran (Tehran)	-	-	Only men	581	24.9	21.3	3.6
3	Mohseni	2005	Iran (Tehran)	-	-	16.2	255	16.1	-	-
4	Radfar	2002	Iran (Khorasan Razavi)	60.9	12.7	4	200	9.5	8	1.5
5	Salehi	2011	Iran (Shiraz)	65.1	12.7	4.8	216	20.4	-	-

### Risk of bias and quality assessment of the included studies

**Fig 2:**
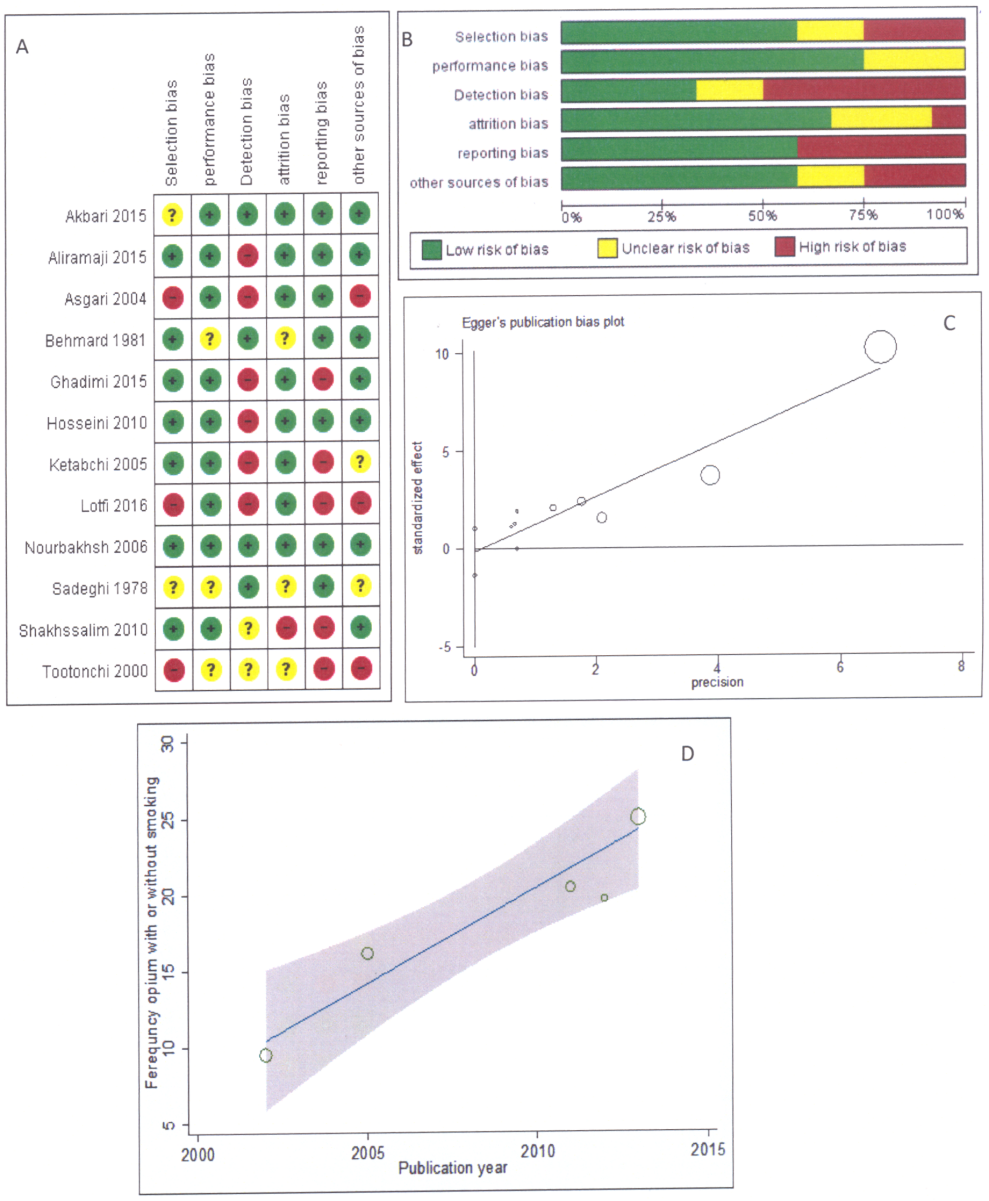
Risk of bias graph, Publication bias and Meta regression, A: review authors' judgments about each risk of bias item for each included study, B: review authors' judgments about each risk of bias item presented as percentages across all included studies, C: Egger funnel plot of just opium use and bladder cancer risk, D: Meta regression analysis for assessment of the heterogeneity scores. Variable of suspected to heterogeneity was publication year.

Fig 2, sections A and B illustrate the risk of bias based on the Cochrane tool. Since the studies were observational, the risks were assessed according to definitions applied in the study by Viswanathan et al. [18]. Selection bias indicated the uniqueness of inclusion/exclusion criteria for the selection of cases/controls or exposed/unexposed groups, and adjustment of the results for potential confounders. Regarding the selection bias, three studies were high risk. Performance bias referred to control of the effect of any un-intended exposure leading to systematic error in the results. Three studies were identified as unclear for such bias.

Regarding attrition bias (non-response rate, missing, drop outs), three studies were categorized as unclear and one study was high risk. Among the primary studies, six studies had high risk of detection bias (blinding outcome/exposure assessment, valid and reliable assessment of outcome/exposure/confounder, and similar time intervals between exposure and outcome in cases and controls), while three had unclear risk. Risk of reporting bias was high in five studies. Other sources of bias such as study design and extreme baseline imbalance were high in three studies and unclear in two other studies. In addition, the quality scores of five cross-sectional studies were higher than 15.5.

### Opium-only consumption and bladder cancer

**Fig 3:**
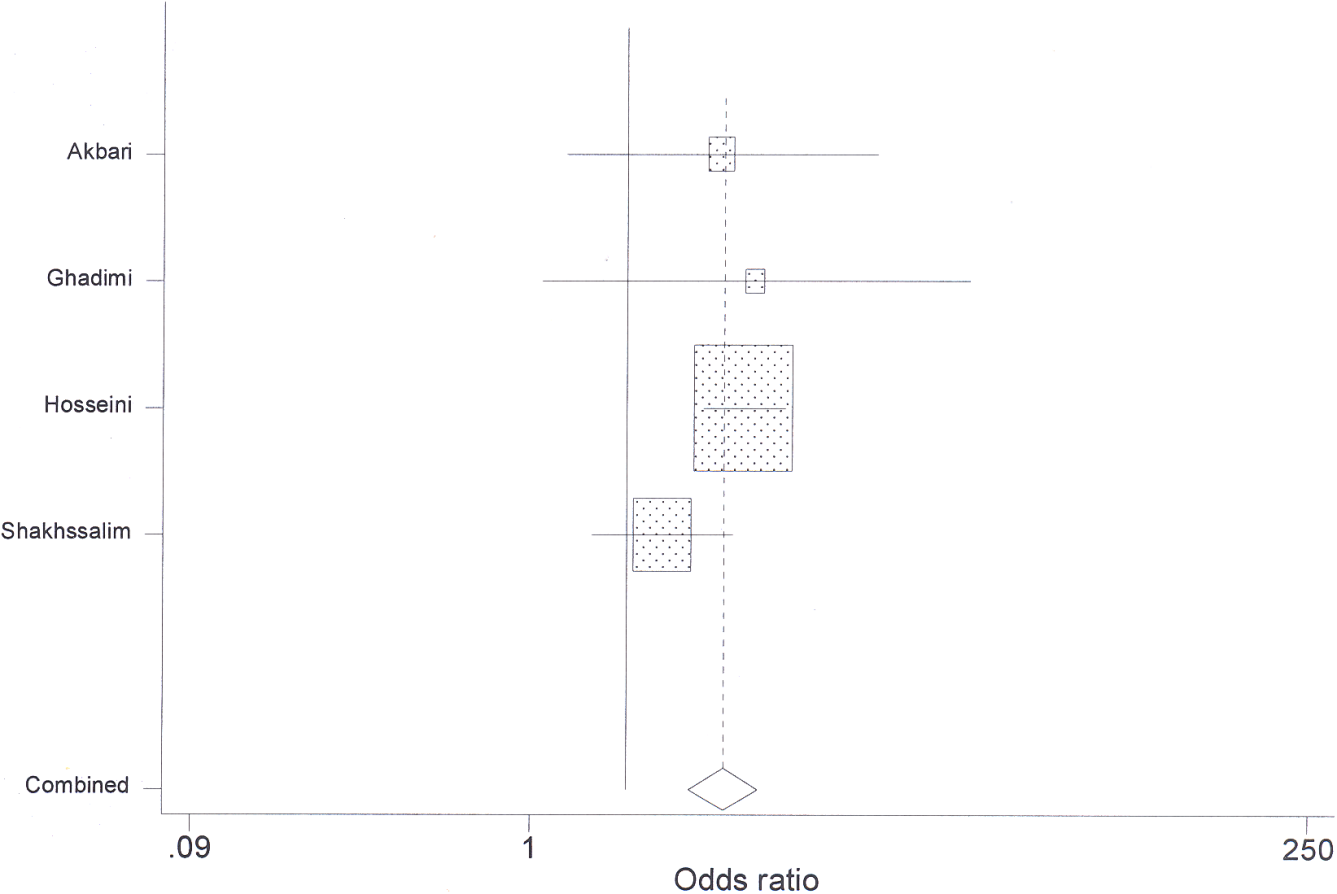
Point and pooled adjusted odds ratios for bladder cancer and opium consumption (in four studies the cigarette smoking-adjusted odds ratios were extracted and combined. Point prevalences of the odds ratios with confidence intervals were illustrated by forest plots. In this plot, the size of each box indicated the weight of each primary study and the crossed lines indicating 95% confidence intervals. At the bottom of each plot, a diamond with a vertical dotted crossed line was designed indicating the pooled prevalence. The horizontal diameter of the diamond indicating the confidence interval of the pooled estimate. Another continuous vertical line in the plot crossed the number one showing the null hypothesis. The 95% confidence interval of each point odds ratio crossing this vertical line considered as non-significant effect).

**Fig 4:**
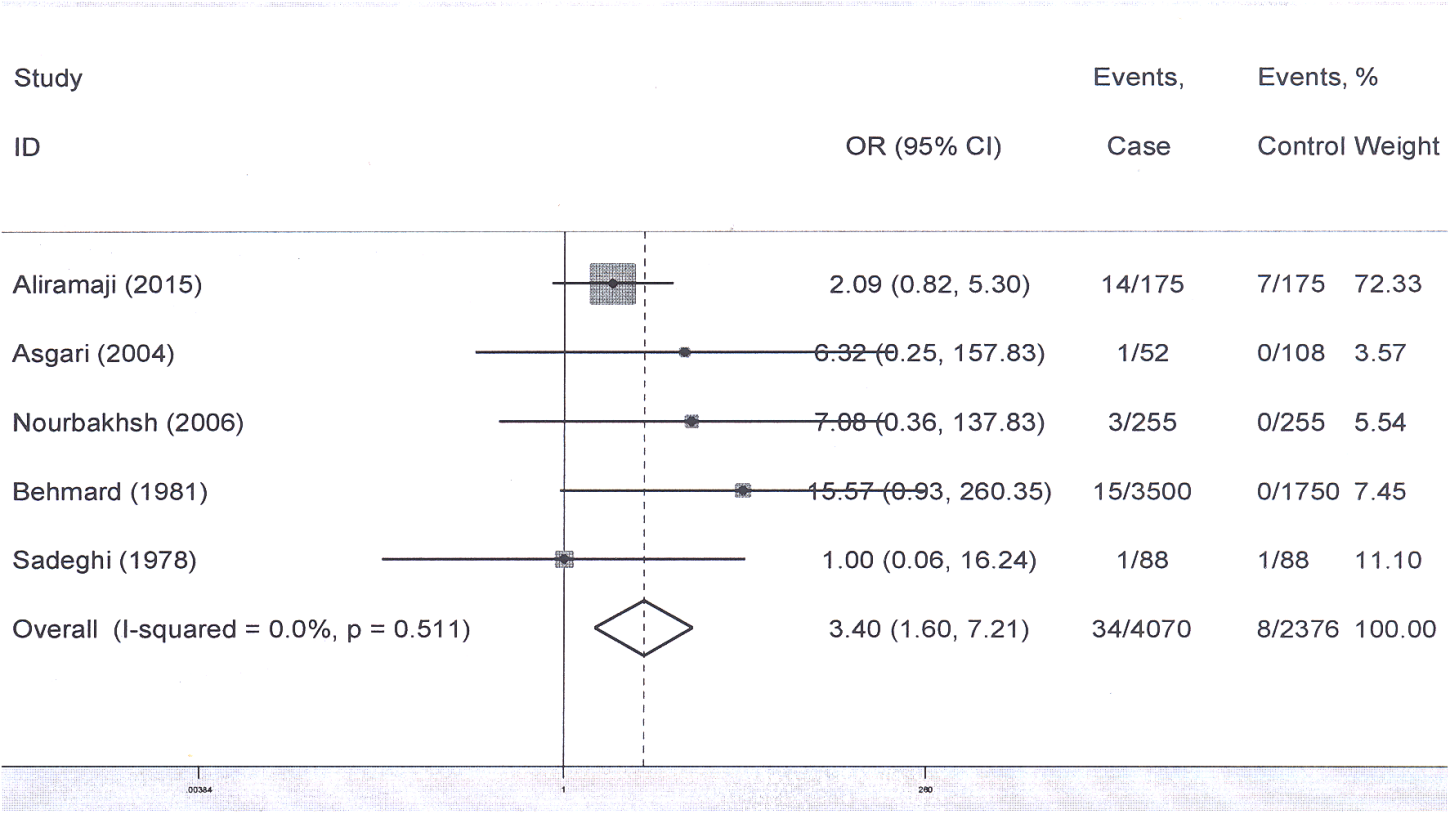
Point and pooled odds ratios for bladder cancer among patients exposed with only opium.

**Fig 5:**
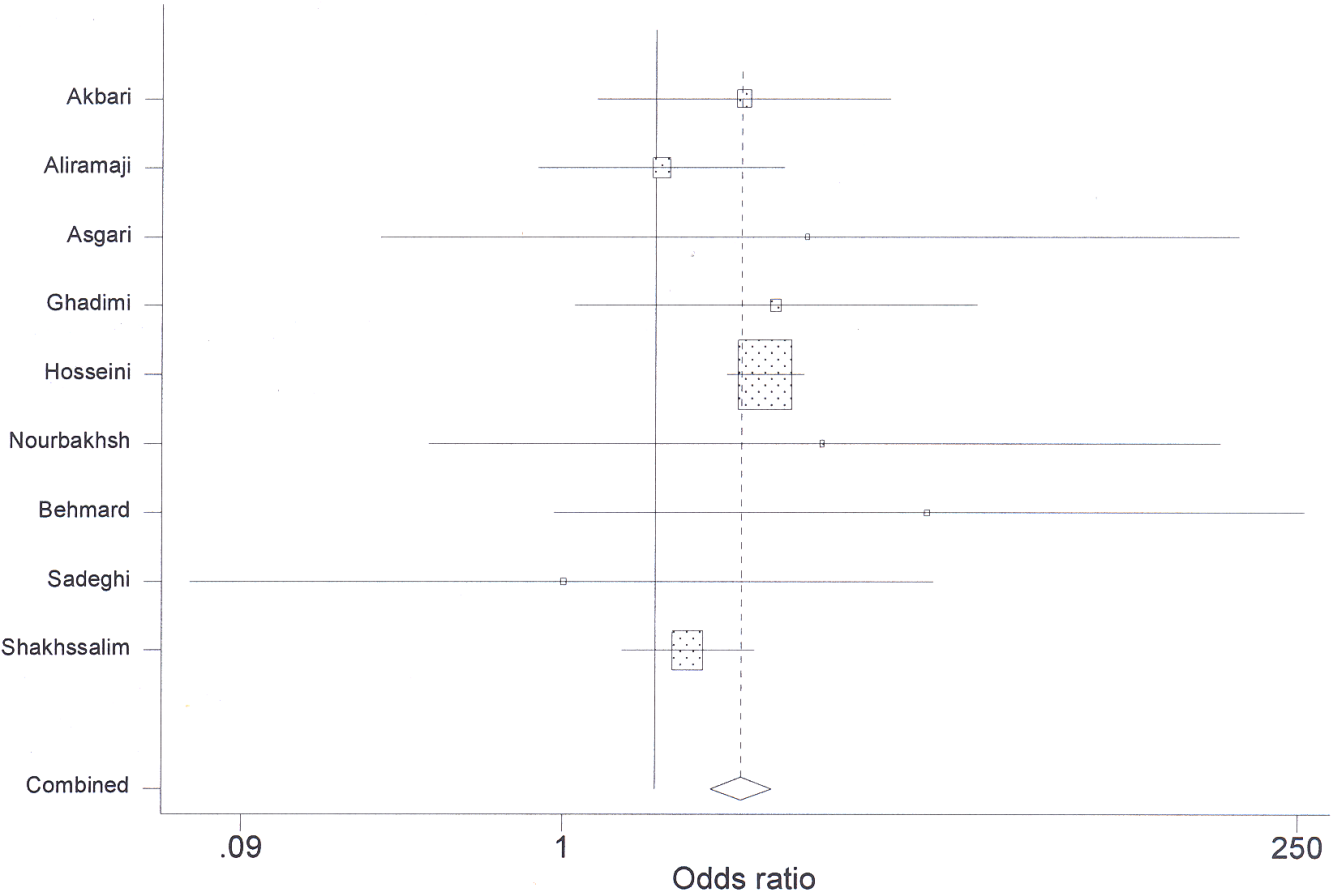
Point and pooled odds ratios (smoking-adjusted OR or OR estimated for non-smokers) with 95% confidence intervals for bladder cancer, since the confidence intervals of the pooled estimates of graphs 3 and 4 were overlapped, the ORs of all nine studies were combined in this plot. Point prevalences of the odds ratios with confidence intervals were illustrated by forest plots. In this plot, the size of each box indicated the weight of each primary study and the crossed lines indicating 95% confidence intervals. At the bottom of each plot, a diamond with a vertical dotted crossed line was designed indicating the pooled prevalence. The horizontal diameter of the diamond indicating the confidence interval of the pooled estimate. Another continuous vertical line in the plot crossed the number one showing the null hypothesis. The 95% confidence interval of each point odds ratio crossing this vertical line considered as non-significant effect).

Out of 12 analytic studies (11 case-control and one cohort), nine studies compared cases and controls regarding just opium use; four of which reported odds ratio for opium use adjusted for other potential confounders including cigarette smoking. Combining the results of these studies using a fixed effect model (Q = 3.9, I-squared = 25%, P = 0.3), showed the pooled odds ratio for pure opium use as 3.9 (95% confidence interval: 3.1–5.1) (Fig 3). Of the five remaining studies, four studies did not find significant results. Combining the results of these five studies using a fixed effect model (Q = 3.3, I-squared: 0%, P = 0.5) estimated the total odds ratio for pure opium use as 3.4 (95% confidence interval: 1.6–7.2) (Fig 4). Applying a fixed effect model, the total odds ratio for all the above nine studies was 3.85 (95% confidence interval: 3.05–4.87) (Fig 5). In addition, no publication bias was observed for the estimates (β = -0.14, P = 0.8) (Fig 2, Section C).

### Concurrent use of opium and cigarettes

In three primary studies, patients with bladder cancer were compared with healthy groups regarding concurrent use of opium and cigarettes. All three studies reported higher frequencies of opium and cigarette consumption among patients. Combining the results of these three studies by random effect model (Q = 9.1, I-squared = 78%, P = 0.01) showed the odds ratio for opium & cigarette use as 5.7 (95% confidence interval: 1.9–16.3).

### Opium or cigarette consumption

In 10 primary studies, cases and controls were compared regarding either cigarette smoking or opium consumption, nine of which observed significant associations. Using a random effect model (Q = 23.4, I-squared = 61.5%, P = 0.005) the pooled odds ratio for this association was estimated as 5.3 (95% confidence interval: 3.6–7.7).

### Dose response effect

The association between bladder cancer and dose of opium used was investigated in two studies (Akbari & Ketabchi). Although both studies showed positive associations, the estimates were not statistically significant.

The effect of the duration of opium consumption on bladder cancer was investigated in five studies (Akbari, ، Ketabchi,، Aliramaji, ، Asgari ،, Hosseini), four of which reported significant results.

In the study carried out by Akbari et al. [11] using variance inflation factor (VIF), no co-linearity was found between opium consumption and cigarette smoking on the effect on bladder cancer.

### Cross sectional studies

Five studies assessed the frequency of opium consumption among 1364 patients with bladder cancer. Results varied between 9.5% in the study carried out by Gavam-Nasiri et al. [32] among 200 patients in 2002 to 24.9% reported by Karbakhsh et al. [29] among 581 patients in 2013. Using random effect model (Q = Heterogeneity chi-squared = 33.2, I-squared = 87.9%, P<0.001), the total prevalence of opium consumption in bladder cancer patients was estimated as 18.06 (95% confidence interval: 12.01–24.09). Out of these cross-sectional studies, two studies reported the frequencies of opium use among cases as small as 3.6% [29] and 1.5% (Radfar), while two studies reported the frequencies of both opium and smoking consumption among patients as 21.3% [29] and 8% [32].

Meta-regression models showed that the date of publication was a significant factor for the heterogeneity of the primary results (β = 1.2, P = 0.011) (Fig 2, section D). Subgroup analysis of the data based on this factor was impossible due to the low number of the primary studies.

## Discussion

According to the controversial reports regarding the real effect of opium consumption on developing bladder cancer, we combined the results of different studies investigating the association between opium and bladder cancer. Our subgroup meta-analysis of the effect of opium addiction showed different estimates for opium-only consumption, opium plus cigarette use, and opium use regardless of smoking status. We also observed that the duration of opium use, but not the dose of opium, was associated with bladder cancer.

There are different mechanisms that are reported for the effect of opium on the development of bladder cancer, Such as exposure to different mutagenic compounds of opium pyrolysates. Such mechanisms are similar to those suggested for tobacco smoking. Moreover, the alkaloids of the opium have reported to increase the time of exposure to cancerogenic agents by inducing urinary retention [12, 42]. Case control studies carried out to investigate the relationship between pure opium addiction and bladder cancer showed less than a four-fold increase in the odds of developing bladder cancer with the use of opium. However, the pooled estimates of the other studies showed higher effects of opium; the risk of bladder cancer among populations with a history of opium consumption was from five to more than 18 times that of those who had not used opium.

Comments made by Mahmoodpoor and Golzari [13] on the Kamangar et al. [12] paper suggested that these observed effects of opium cannot be generalized to all populations because the differences between populations regarding genetic and other factors. They could not combine the results by meta-analysis due to different risk factors of cancers as well as highly correlation between cigarette smoking and opium consumption. Therefore, it is better to estimate the effect of opium independently from tobacco smoking.

Considering the proven harmful effect of cigarette smoking as a risk factor increasing the risk of bladder cancer more than two folds [43] by N′-nitrosonornicotine, 4-(methylnitrosamino)-1-(3-pyridyl)-1-butanone and mentol [44], the excessive effect observed in these studies can be mostly due to this factor. In other words, opium can cause bladder cancer by at least two pathways. The first is by the mechanism induced by cigarette smoking as a confounder. The other pathway is unknown in many studies and may be other potential or risk factors such as occupational hazards, genetic, environmental, or anthropometric factors. In addition, the potential role of cigarette smoking as an effect modifier should not be ignored. In the study conducted by Karbakhsh et al. [29], the age at diagnosis of bladder cancer was significantly lower in patients who were dependent on both cigarettes and opium compared with that among patients did not use opium or cigarettes.

It should be noted that the results reported by the cross-sectional and case-control studies might be prone to biases. The temporal relationship is not clear in such studies; therefore, the observed associations might be true or reverse relationships. For example, patients with bladder cancer may use opium products as analgesics or sedatives. It is better to investigate the associations between opium use and cancer by conducting longitudinal studies.

Primary studies entered into the current meta-analysis had been conducted using different methodologies. For example, matching the case-control groups as well as the confounders adjusted during the odds ratio estimations were not the same among these primary studies. In addition, it was not possible to provide the odds of developing bladder cancer among opium-only users. On the other hand, all of the primary studies were conducted in Iran and no English-written research regarding the association between bladder cancer and opium consumption was identified during our comprehensive search. Moreover, in the current mea-analysis, we investigated the association between opium use and bladder cancer and did not consider the effect of the opium consumption method. It should be taken into consideration by the future studies. These studies should also assess the differences between the crude and adjusted associations to explain the different pathways of opium effect including the cigarette pathway.

The results of this meta-analysis can provide important information for the general population to protect themselves against a proven risk factor for one of the highly virulent cancers. People should not only prevent and stop smoking habits, but also avoid other behavioral risk factors such as opium consumption.

Finally, Considering the increasing trend of opium consumption among populations especially in countries such as Iran which is located in a critical gateway of opium substances, our study provided credible evidence for policymakers to establish immediate strategies for preventing this behavior in the communities such as educational programs, restrictions, and appropriate penalties.

## Conclusion

In conclusion, our meta-analysis showed that opium consumption is independent potential risk factor for bladder cancer. Therefore, it could be a risk factor for other cancers. Further studies are recommended to investigate the independent effect of opium and its dose effect on developing bladder cancer, as well as the interaction between opium and cigarette use. We also recommend that similar studies are carried out in other countries.

## Supporting information

S1 File PRISMA checklist. (DOC)
